# Microglia and Astrocytes in the Lateral Vestibular Nuclei of Mice after Vestibular Stimulation

**DOI:** 10.32607/actanaturae.27673

**Published:** 2026

**Authors:** I. B. Mikheeva, N. S. Zhuikova, D. A. Fedorov, O. Yu. Antonova, L. L. Pavlik, V. I. Arkhipov

**Affiliations:** Institute of Theoretical and Experimental Biophysics, Russian Academy of Sciences, Pushchino, Moscow Region, 142290 Russia

**Keywords:** vestibular stimulation, lateral vestibular nuclei, Deiters’ neurons, microglia, astrocytes, gene ex pression, GFAP, Aif1

## Abstract

BALB/C mice were subjected to vestibular loading (rotation in individual containers at a speed of 80 rpm) for 8 h. As a result of this loading, the animals exhibited a decrease in horizontal and vertical locomotor activity, which returned to the control levels after 5 days. An immunohistochemical study of mi croglia and astrocytes in the lateral vestibular nuclei (LVN) revealed elevated levels of protein markers for astrocytes (GFAP) and microglia (Aif1) one hour and 5 days after the stimulation. These changes were indic ative of a gradual development of neuroinflammation in the LVN, which lasted for at least 5 days. Microglia, which appeared in branched shape in control animals, acquired an amoeboid reactive shape after vestibu lar loading. Moreover, expression of the genes coding for these proteins remained at the control level one hour after the stimulation and showed a reduction after 5 days. It is assumed that such a decrease helps re solve the neuroinflammation, preventing it from becoming chronic. Neuroinflammation in the acute phase is known to play a protective role and is required for plastic rearrangements of neuronal and glial networks. Transition to the chronic phase results in neuronal damage. The results of this study would allow one to de termine the period when it is reasonable to use anti-inflammatory therapy to mitigate damage. The applied model of vestibular stimulation allows one to solve problems when studying plastic rearrangements in the brain structures of the vestibular system under high-intensity sensory load.

## INTRODUCTION

The vestibular system consists of a peripheral and a central component. The peripheral vestibular system, located within the inner ear, comprises three semicir cular canals that detect rotational movements of the head, and otolith saccules that sense gravity and lin ear acceleration. The input from the peripheral ves tibular apparatus is transmitted to the brain via the vestibular nerve. The central vestibular system, pre dominantly located in the brainstem and cerebellum, processes this information and interacts with the oc ulomotor nucleus, ensuring gaze stability during head motion. As a whole, the vestibular system helps main tain postural equilibrium and stabilize vision by mon itoring head and body movements, as well as spatial orientation. Dysfunction of the vestibular system may cause sensory disturbances, including balance disor ders, vertigo, and blurred vision [1].

The lateral vestibular nucleus (LVN), also known as Deiters’ nucleus, is one of the four principal ves tibular nuclei located within the medulla oblongata and pons. Deiters’ nucleus plays a pivotal role in the maintenance of posture and equilibrium, as it re ceives input from the vestibular apparatus, the cer ebellum, the spinal cord, and the reticular formation [2, 3, 4, 5]. The efferent connections of the LVN include the lateral vestibulospinal tract, which is the most significant pathway, as well as connections with the cerebellum (providing feedback for movement coor dination), oculomotor nuclei, thalamus, and neocor tex. Functionally, the LVN acts as the primary source of vestibulospinal influences, contributing to postural maintenance, coordination of movement upon changes in body position, and stabilization of the oculomotor response during head motion. Hence, the LVN inte grates vestibular, proprioceptive, and cerebellar signals to ensure postural stability and motor coordina tion [2]. Lesions in the LVN can be accompanied by ataxia (impaired postural balance), loss of extensor muscle tone, and nystagmus (involuntary eye move ments). Vestibular system dysfunction often derives from stroke, neurodegenerative disorders, tumors, or intoxication. Animal experiments indicate that the vestibular system undergoes structural and functional changes after labyrinthectomy (as a model of acute neurodegeneration), upon spinocerebellar ataxia (neu ronal degeneration) or multiple sclerosis (axonal de myelination) [6, 7, 8].

Neuroinflammation is one of the results observed upon various types of brain injury, including unilater al vestibular hypofunction. It has been demonstrated that neuroinflammation contributes to the functional recovery of neural tissue [9]. Reactive microglia, the key mediators of neuroinflammation, may perform a critical function in the vestibular nucleus by protect ing the affected area against injury. Expression of the inflammatory marker (tumor necrosis factor-al pha (TNF-α)), as well as the neuroprotective markers (nuclear factor kappa B (NF-κB) and manganese su peroxide dismutase (MnSOD)), has been shown to be upregulated during acute inflammation in the vestib ular nuclei after unilateral vestibular deafferentation [10]. Upon activation, microglial cells are recruited to the site of the infection or CNS injury; they un dergo phenotypic transformation and release several pro-inflammatory mediators that facilitate the clear ance of cell debris and dead cells. Microglial activa tion is observed in patients with Parkinson’s disease, Alzheimer’s disease, multiple sclerosis, and other neu rodegenerative disorders [11].

In general, microglia are essential for maintain ing brain homeostasis: they eliminate damaged cells upon inflammation, are involved in neurotransmitter metabolism, and control the extracellular ion balance. Iba1 (ionized calcium-binding adapter molecule 1), a 17 kDa calcium-binding protein encoded by the AIF1 (allograft inflammatory factor 1) gene, is a ubiq uitous marker for microglia [12]. This protein plays a crucial role in cytoskeletal reorganization and immune responses; its expression is upregulated upon neuroin f lammation (e.g., in patients with Alzheimer’s disease and multiple sclerosis) [12, 13].

Another type of glial cells, astrocytes, likewise play a substantial role in maintaining brain homeo stasis. Astrocytes supply neurons with metabolites and growth factors, support synaptogenesis, and regulate the extracellular concentration of ions and neurotransmitters. They are key players in neuro inflammation, having both protective and detrimen tal effects. Furthermore, they are implicated in the pathogenesis of neurodegenerative disorders, includ ing Alzheimer’s disease and amyotrophic lateral scle rosis [14, 15]. Astrocytes are among the first cells (or even the first ones) to respond to a CNS injury. The morphological transformation of reactive astrocytes involves hypertrophy of their cell bodies and process es [16]. GFAP (glial fibrillary acidic protein), a key structural protein in astrocytes, is a glial cell damage marker [17, 18].

In this study, we investigated the changes in the astrocytic and microglial states within Deiters’ nuclei following increased vestibular load over time. Our f indings may help identify conditions in which thera peutic interventions following damage to the vestibu lar nuclei can be used.

## EXPERIMENTAL

**Animals**

Experiments were conducted using adult male BALB/c mice aged 19–20 weeks and weighing 30 g. The animals were housed under standard vivarium conditions with ad libitum access to food and wa ter. All the experiments were approved by the Ethics Committee for Animal Experiments and the Biological Safety and Bioethics Commission of the Institute of Theoretical and Experimental Biophysics, Russian Academy of Sciences (Protocol No. 8/2024 dated March 18, 2024), and were carried out in com pliance with Directive 2010/63/EU of the European Parliament and the Council on the protection of ani mals used for scientific purposes.

**Vestibular stimulation**

Once the animals were obtained from the vivarium, they were habituated to handling by the experimenter for three days. Next, they were divided into three groups: the control group (Group C) and two experimental groups, S0 and S5 (12 mice per group;* n *= 12). Each animal in the experimental groups was placed into a container (7 × 12 cm) and subjected to vestibular stimulation (rotation in the vertical plane at 80 rpm for 8 h). Control animals were kept in identical containers next to the apparatus without stimulation for 8 h.

**Behavioral tests**

Open-field behavioral tests were conducted immediately after the stimulation. Each mouse was placed in the center of an open 50 × 30 cm arena divided into 10 × 10 cm squares. The number of rearing events (vertical activity) and the number of square crossings (horizontal locomotor activity) over a 10-min period were recorded.

**Real-time RT-PCR**

After the behavioral tests (1 h after the cessation of vestibular stimulation for eight animals from groups C and S0; 5 days for the animals from group S5), the mice were euthanized by cervical dislocation. The brain area containing vestibular nuclei was removed. The resulting tissue samples were homogenized in a denaturing buffer containing guanidine isothiocyanate (Sigma, USA). Total RNA was extracted from the homogenized brain tissue by phenol–chloroform extraction. RNA was purified using a DNaseI kit (RNase-free, New England Biolabs, USA), according to the manufacturer’s protocol. Concentration of the isolated RNA was determined spectrophotometrically; sample quality was assessed by 1% agarose gel electrophoresis. Reverse transcription was performed in compliance with the standard protocol provided by the reverse transcriptase manufacturer (Fermentas, USA). Real-time quantitative PCR was performed on a DT-322 amplifier (DNA-Technology, Russia) using the SYBR Green intercalating dye (Invitrogen, USA) and the β-actin gene as a reference gene. The mRNA levels in the mouse vestibular nuclei were quantified based on the threshold cycle (Ct) detected by the amplifier, followed by calculations using the 2^(-ΔΔCt) method. The quality and molecular weight of the PCR products were confirmed by 3% agarose gel electrophoresis. Amplification was conducted using gene-specific primers for glial cell marker proteins: GFAP for astroglia and AIF1 for microglia (*Table 1*).

**Table 1:** The nucleotide sequence of the primers used in our study

Gene	Primers, nucleotide sequence	Length of the product, bp
Actb	**F** CTTCTTGGGTATGGAATCCTG	190
	**R** CTTGATCTTCATGGTGCTAGG	
AIF1	**F** TGGAGGGGATCAACAAGCAAT	71
	**R** AAGTTTGGACGGCAGATCCTC	
GFAP	**F** TGGTATCGGTCTAAGTTTGCAG	88
	**R** GTCGTTAGCTTCGTGCTTGG	

**Immunohistochemistry**

Four animals from each group (groups C, S0, and S5) were anesthetized with a xylazine–Zoletil mixture (tiletamine– zolazepam) at a dose of 50 mg/kg and transcardially perfused with cold phosphate-buffered saline (PBS) and then 4% paraformaldehyde (PFA). The brains were removed and placed into paraformaldehyde for 24 h. Floating fixed brain sections, 50 μm thick, were prepared using a vibratome (VIBRATOME 100; IMEB). The sections were then stained with primary antibodies specific to the microglia marker Iba1, the astrocyte marker GFAP, and the neuronal nuclear marker (NeuN) (1 : 1,000). The following secondary antibodies were used: goat anti-rabbit (Alexa Fluor 594) a11007 for Iba1; goat anti-chicken (Alexa Fluor® 647) ab150171 for GFAP; and goat anti-guinea pig (Alexa Fluor® 405) ab175678 for NeuN (1 : 500).

The specimens were mounted under coverslips on ProLong Glass Antifade Mountant (Invitrogen, USA).

**Quantitation of the histological changes**

Microscopy examination was conducted using a TCS SP5 confocal microscope (Leica, Germany) equipped with a 40× oil immersion lens (numerical aperture, 1.25) to acquire Z-stacks (8–18 optical sections with a step size of 3 μm). All the images were captured at identical acquisition parameters as 16-bit images sized 1024 × 1024 pixels. The Fiji ImageJ v.1.54p software (https://doi.org/10.1038/nmeth.2019) was used for image processing. The maximum intensity projections were generated using the Extended Depth of Field plugin (https://bigwww.epfl.ch/demo/edf/).

Only the areas containing Deiters’ neurons were analyzed. Regions sized 200 × 200 μm were selected for each image, and at least 25 such regions were analyzed per group. The “threshold” function was applied in order to efficiently distinguish between the GFAP- or Iba1-positive areas and background staining. The percentage of the image area with a fluorescence intensity above the threshold was calculated using the built-in “measure” function. The data are reported as a mean ± standard deviation. Statistical analysis was performed using the two-sample Student’s *t*-test.

**Statistical analysis**

The statistical analysis was performed using the GraphPad Prism software (version 8.0.2.263). Mouse behavior was evaluated using the non-parametric Mann–Whitney U test. Statistical significance was set at *p *≤ 0.05. Data are presented as the mean ± SEM. For immunohistochemical and RT-PCR data, the normality of sample distributions was verified using the Shapiro–Wilk test. The significance of intergroup differences was assessed using the one-way analysis of variance (ANOVA), followed by Tukey’s multiple comparison test. The Kruskal–Wallis test, followed by Dunn’s post-hoc multiple comparison test, was applied for non-normal distributions.

## RESULTS AND DISCUSSION

**Behavior**

**Table 2:** Animal behavior in the open-field test after vestibular stimulation

Animal group	Number of square crossings in the openfield test	Number of rearings	Total duration spent grooming
Control (C) (n = 12)	146.3 ± 3.7	44.4 ± 2.4	60.8 ± 5.0
C0 (n = 12)	76.1 ± 7.6^*^ (p < 0.0001)	0.6 ± 0.3^*^ (p < 0.0001)	356.6 ± 15.7^*^ (p < 0.0001)
C5 (n = 12)	142.4 ± 15.0^#^ (p = 0.0031)	38.4 ± 4.8^#^ (p < 0.0001)	131.5 ± 22.2^*,#^ (p = 0.0014, p < 0.0001)

^*^ – relative to group C;

^#^ – relative to group S0.

The animals experienced significant stress during vestibular stimulation. After removal from the containers, they showed signs of physical fatigue caused by prolonged exposure to an unusual environment. The open-field test revealed a substantial decline in locomotor activity 1 h following vestibular stimulation: the number of square crossings dropped almost twofold, while the number of rearing events decreased severalfold. Five days after stimulation, both the horizontal and vertical activity levels remained not different from the control values (*Table 2*).

The reduction in locomotor activity expected immediately after vestibular loading had normalized within several days, as demonstrated by the tests conducted 5 days post-stimulation. The intensified grooming behavior observed during this period can be attributed to a vigilance reaction upon contact with the experimenter due to memories of a stressor. BALB/c mice are known to exhibit high levels of anxiety; they are very emotional and vulnerable, and they experience difficulty coping with stress [19, 20].

The changes in animal behavior after vestibular stimulation, including a reduced locomotor activity level and intensified grooming, can be attributed not only to acute stress, but also to long-term changes in the LVN. To test this hypothesis, we performed a immunohistochemical analysis to assess the state of microglia and astrocytes, which are the key cells involved in the neuroinflammatory response.

**Immunohistochemistry**

**Fig. 1:**
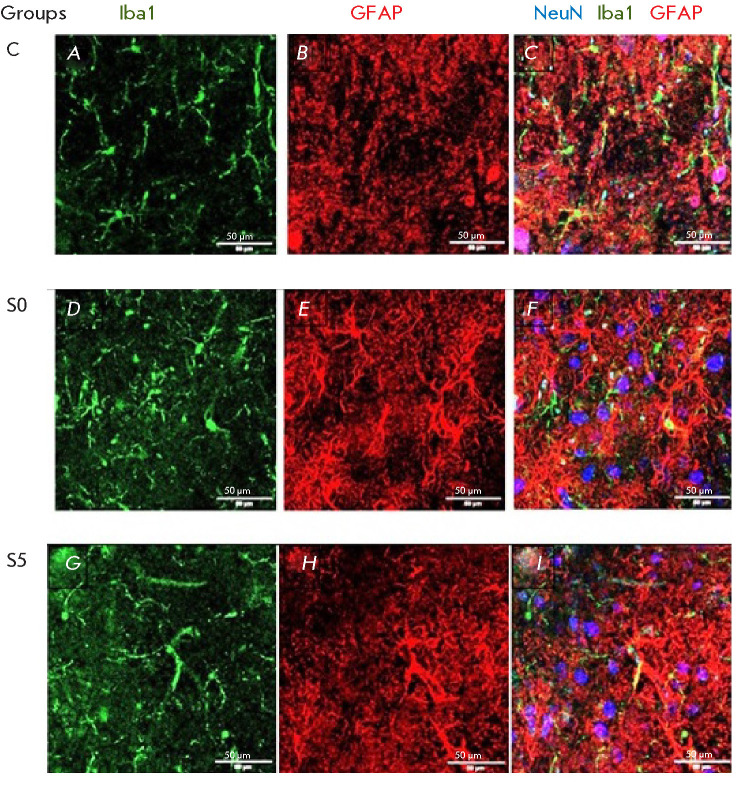
The effect of vestibular stimulation on the morphology of microglia and astrocytes. The green channel was used to identify microglia (Iba1-positive cells); the red channel, for astroglia (GFAP-positive cells); and the blue channel, for neurons (NeuN-positive neuronal nuclei). Representative micrographs of sections of the lateral vestibular nuclei in the animals in Group C (*A*–*C*), Group S0 (*D*–*F*), and Group S5 (*G*–*I*). Scale bar, 50 μm

Immunohistochemical staining revealed that in the control animals, microglial cells remained uniformly distributed in the lateral vestibular nucleus and kept a well-defined cell body with branched processes (*Fig. 1A*). In the animals in group S0, the increased vestibular load altered the morphology of microglia: their cell bodies now presented either a rounded spherical shape and reduced processes, or a rod-like morphology (*Fig. 1D*). These morphological changes contribute to increased motility and are indicative of the activated state of microglia [21, 22].

**Fig. 2:**
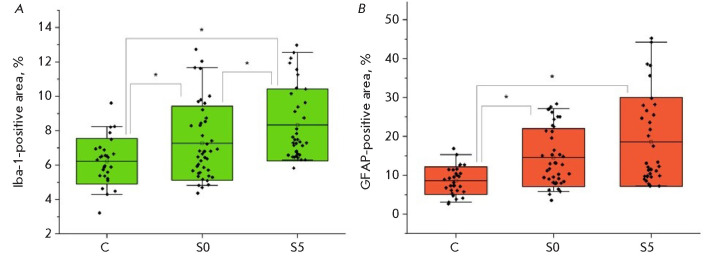
Immunohistochemical staining of the (*A*) Iba1 (green) and (*B*) GFAP (red) proteins in lateral vestibular nuclei. The relative area of immunohistochemical staining in the lateral vestibular nuclei in the animals of Group C, one hour (Group S0) and 5 days (Group S5) after vestibular loading

Rod-like microglia are typically a feature of the early stages of neurodegenerative disorders and injuries [23]. The astrocytes in the lateral vestibular nuclei of the animals after stimulation also underwent characteristic changes. Activated cells were observed in group S0 (*Fig. 1E*); as demonstrated previously [24, 25, 26], they had a hypertrophied cell body and thickened primary branches with reduced peripheral processes. Since astrocyte activation leads to the formation of denser intermediate filaments that contribute to structural rigidity [27], we detected an elevated GFAP content in the animal groups S0 and S5 (*Fig. 2B*).

**Real-time RT-PCR**

**Fig. 3:**
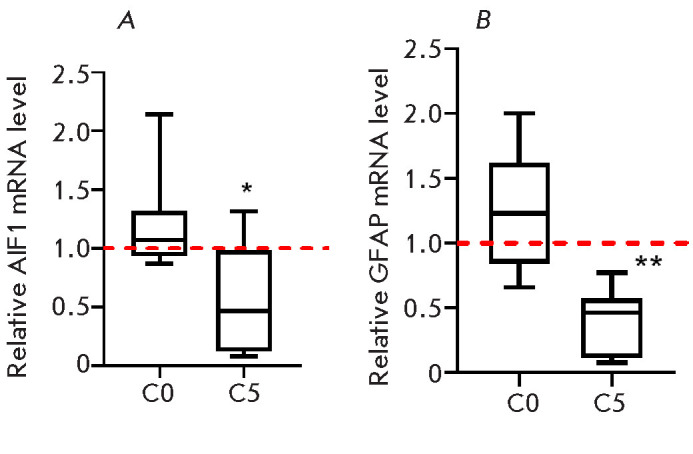
The mRNA level of proteins, markers of microglia (*A*), and astrocytes (*B*) one hour (Group S0) and 5 days (Group S5) after vestibular loading relative to the control animals. Statistical intergroup differences were determined using one-way analysis of variance followed by Dunnett’s multiple comparison test. *p < 0.006; **p < 0.0001 relative to the control

The Iba1 and GFAP mRNA levels in the lateral vestibular nuclei were assessed 1 h and 5 days after the stimulation. Similar patterns in the expression of marker proteins for both microglia and astrocytes were observed: the mRNA levels in the animals in group S0 did not deviate from their control values, while they dropped 5 days after stimulation in the animals in group S5 (*Fig. 3*).

Although the GFAP mRNA level 1 h after the stimulation had remained unchanged, the content of this protein in the lateral vestibular nuclei was elevated (*Fig. 1E*, *Fig. 2B*). Five days after the stimulation, the GFAP levels within the nuclei remained elevated (*Fig. 1H*, *Fig. 2B*), while the mRNA level of this protein was substantially reduced. Expression of the* GFAP *gene is regulated by numerous transcription factors, including various protein kinases and other signaling molecules [28]. However, the regulation of the synthesis of this protein is also exceptionally complex and versatile [15]. Therefore, there is often a misalignment between the dynamics of *GFAP *gene transcription and protein levels under various impacts (in particular, upon different types of neuroinflammation) [29]. It can be assumed that neuroinflammation does develop in the lateral vestibular nuclei under our experimental conditions, as was further evidenced by microglial activation. The level of the Iba1 protein marker for microglia was elevated in the animals in groups S0 and S5 compared to the controls (*Fig. 2A*), although expression of both the *AIF1 *and *GFAP *genes was reduced in mice in group S5 (*Fig. 3A*). This misalignment between the AIF1 and Iba1 RNA levels is also attributable to the complex regulation of the transcription and translation of this protein in the brain. Findings demonstrating the regulatory role of HIF-1α (hypoxia-inducible factor-1α) in the mechanisms of innate immune memory and MPTP-induced Parkinsonian pathology are an example of such discordance [30]. This factor was found to stabilize the Iba1 protein under hypoxic conditions without altering the *AIF1 *expression level.

## CONCLUSIONS

Our study suggests that vestibular stimulation elicits a complex response in the LVN, encompassing both behavioral and cellular changes. The observed signs of neuroinflammation – such as microglia and astrocyte activation, as well as misalignment between the protein and mRNA expression levels — are consistent with the neuroplasticity mechanisms and pathological processes that have been described previously. Neuroinflammation in the lateral vestibular nucleus progresses through phases such as acute microglial activation, a peak cytokine response, all followed by resolution or progression to a chronic state. It had been demonstrated recently that a transient inflammatory response occurring in the deafferented vestibular system is essential for the formation of intrinsic plasticity, which facilitates functional recovery. Reactive microglia in the vestibular nucleus may play a critical role by protecting the affected area against chronic inflammation and contributing to the long-term survival of afferent vestibular neurons [9]. However, it is well-known that chronic activation of microglia and astrocytes may result in excessive inflammation, neuronal damage, and exacerbation of neurodegeneration [31].

Hence, it is clear that anti-inflammatory neuroprotection therapy must be administered within specific temporal windows to prevent the escalation of neuroinflammation into a chronic state. The observed reduction in the *GFAP *and *AIF1 *gene expressions in the vestibular nuclei of the animals 5 days after vestibular stimulation was likely to do with a protective mechanism facilitating the resolution of neuroinflammation and blocking its progression to a chronic form. The model of vestibular stimulation suggested in this study allows one to investigate plastic rearrangements in the vestibular structures of the brain upon highintensity sensory load.
